# Interactive lectures: Clickers or personal devices?

**DOI:** 10.12688/f1000research.6207.1

**Published:** 2015-03-12

**Authors:** Lesley J. Morrell, Domino A. Joyce

**Affiliations:** 1 School of Biological, Biomedical & Environmental Sciences, University of Hull, Kingston-upon-Hull, HU6 7RX, UK

**Keywords:** Classroom response system, Interactive teaching, Interactive lecture, Personal response system

## Abstract

Audience response systems (‘clickers’) are frequently used to promote participation in large lecture classes, and evidence suggests that they convey a number of benefits to students, including improved academic performance and student satisfaction. The limitations of these systems (such as limited access and cost) can be overcome using students’ personal electronic devices, such as mobile phones, tablets and laptops together with text message, web- or app-based polling systems. Using questionnaires, we compare student perceptions of clicker and smartphone based polling systems. We find that students prefer interactive lectures generally, but those that used their own device preferred those lectures over lectures using clickers. However, device users were more likely to report using their devices for other purposes (checking email, social media etc.) when they were available to answer polling questions. These students did not feel that this distracted them from the lecture, instead, concerns over the use of smartphones centred around increased battery usage and inclusivity for students without access to suitable technology. Our results suggest that students generally preferred to use their own devices over clickers, and that this may be a sensible way to overcome some of the limitations associated with clickers, although issues surrounding levels of distraction and the implications for retention and recall of information need further investigation.

## Introduction

Audience response devices, also known as electronic voting systems or ‘clickers’ have a strong body of evidence supporting their use in Higher Education ( Caldwell, 2007; Kay & LeSage, 2009; reviewed in Keough, 2012). Clickers have been found to be useful for engaging students in large lecture classes, promoting participation, facilitating reflection and formatively (and anonymously) testing understanding. Lecturers can adjust their teaching in real time in response to difficulties that students may be having with particular concepts, to promote understanding. Further benefits of using clickers include increased attendance, improved attention spans and positive outcomes such as higher levels of academic performance and student perception of satisfaction (reviewed in Barnett, 2006; Kay & LeSage, 2009; Keough, 2012; Salemi, 2009; Sutherlin *et al.*, 2012).

However, there are a number of drawbacks to using clickers. There is a time investment involved in preparing interactive lectures that may not pay off if access to hardware is limited because of lecturer demand versus investment at an institutional level. Additionally, handing out and collecting hardware can eat into teaching time ( Dunn *et al.*, 2013; King & Robinson, 2009). However, many students now carry with them personal devices such as mobile phones, tablets and laptops that allow for similar interactive lectures styles using these technologies ( Brett, 2011; Dahlstrom & Bichsel, 2014). Text-message based interaction has already received attention in the literature ( Voelkel & Bennett, 2014). Text-based systems allow similar multiple-choice responses to lecturer questions, but have the added benefit of allowing students to post questions to a ‘text-wall’ or similar, something that is not possible with most clickers. The drawbacks of a text-based system, however, centre around the cost to the student ( Voelkel & Bennett, 2014): each message carries a financial obligation on the part of the student (although some students will have bundles offering unlimited texts, many will have a limited number and some will pay per message). This additional cost to the students may be considered unacceptable, particularly where students pay fees for their education.

Web-based (or app-based) response systems are increasingly available and carry with them the benefits of both clickers and text-message systems ( Dunn *et al.*, 2013), with options for multiple choice, numerical answer and text-based questioning. Their use is less well studied, tending to focus on the evaluation of particular systems (e.g. Dunn *et al.*, 2013; Méndez & Slisko, 2013; Voelkel & Bennett, 2014; Wash, 2014). Web-based systems that connect via a University’s wireless network will carry no additional cost to the student. Possible drawbacks centre on a student’s likelihood of distraction, particularly by social networks while online ( Dunn *et al.*, 2013). This could pose a problem because there is evidence to suggest that individuals are not as skilled at multi-tasking as they might think ( Sanbonmatsu *et al.*, 2013). Multi-tasking is particularly difficult when the tasks are similar (i.e. use the same cognitive channels), such as simultaneously viewing lecture slides on a screen and reading social network status updates ( Rubinstein *et al.*, 2001; Trafton & Monk, 2007). Text-message interruptions to a lecture can reduce learning ( Conard & Marsh, 2014; Rosen *et al.*, 2011), and laptop usage ( Fried, 2008), particularly long browsing sessions ( Grace-Martin & Gay, 2001) and behaviours unrelated to academic work ( Gaudreau *et al.*, 2014), such as using Facebook can reduce academic (test) performance ( Junco, 2012; Junco & Cotten, 2012). Distraction or disruption can also result in students being less able to apply their knowledge flexibly in new situations ( Foerde *et al.*, 2006). In addition, recent work suggests that checking social networks during lectures can also be distracting to other students for whom the screen is visible, and can have implications for information retention, measured by lower test scores ( Sana *et al.*, 2013).

Here, we report the results of a web-based system trial, using students’ own devices, and compare the costs and benefits to both the use of standard clickers in lectures, and to lectures with a similar level of interaction but no technology. We use paper-based questionnaires and focus groups to analyse students’ perception of enjoyment, understanding, ease of use and distraction in the three types of lectures.

## Methods

We carried out the trial in two level 5 (year 2 of a standard UK 3 year Bachelor degree) modules in the School of Biological, Biomedical and Environmental Sciences at the University of Hull, in lectures by two different lecturers (Behavioural Ecology, LJM, 104 registered students; Evolutionary Biology, DAJ, 38 registered students). There was some overlap in the students taking the modules. In each module, at least one lecture used institutionally-held clickers (Turning Technologies Response Card RF with Turning Point; ‘clicker lectures’), at least one used an app-based response system (eInstruction Flow ( http://www.einstruction.eu/downloads/)), since acquired by Turning Technologies; ‘device lectures’), and at least one used ‘hands-up’ or ‘shout-out’ interaction (‘no-technology lectures’). We chose the eInstruction app-based system because it was free for the students to use, being costed on a per-instructor basis. Also, to avoid any potential disadvantage to students who were unable or unwilling to download the app for the ‘device lectures’ (available for Android and iPhone), we purchased a small number of “crickets” – clicker hardware devices that are integrated, allowing both the app and clickers to be used simultaneously. Students were advised in advance to download the app, but those that did not, or did not possess suitable personal technology, were able to collect a cricket at the start of the device lectures.

Between four and eight multiple choice questions were devised for each lecture, and the type of lecture assigned randomly throughout the semester. Lecture slots were either 50 minutes (Evolutionary Biology) or 110 minutes with a break mid-way through the session (Behavioural Ecology). Students answered the questions using the technology available in that particular lecture. After the final lecture, students were asked to complete a paper-based questionnaire ( Supplementary Material) asking a range of questions about the technology they possessed, their perception of their enjoyment, understanding and informativeness of the different types of lecture, their likelihood of distraction and preferred approach to interactive lectures. Students who used their own technology to answer the questions in the device lectures completed a version of the questionnaire comparing all three types. Students who did not use their own technology answered a modified version of the questionnaire comparing the no-technology lectures with the clicker lectures. We used two follow-up semi-structured focus group interviews with ten participants in total (one group of eight, one group of two) to obtain a more nuanced understanding of student perceptions of using their own technology in lectures. Focus groups primarily concentrated on the distraction element of the students using their own technology as response systems, and all participants were volunteers.

### Data analysis

The proportions of device and clicker users giving particular responses were compared using proportion tests (prop.test in Rv2.13.0; R Development Core Team, 2011). Exact binomial tests were used to assess whether the proportion of participants giving a particular response was greater than 0.5.

### Ethical considerations

The School of Biological, Biomedical and Environmental Sciences and the Faculty of Science and Engineering ethical approval committees approved the project and questionnaires prior to any work commencing. Questionnaire data (including student contact details) was input and anonymised by student interns, who also carried out and transcribed focus group interviews. Lecturing staff were unaware of student identity in data analysis. Consent information ( Supplementary Material) was included with the questionnaire: participation was voluntary and students were free to withdraw at any time.

## Results

Demographic data Demographic data (age, gender, degree programme, types of devices owned and used in the trial) for all students completing the questionnaire. Please see Contents_of_data_files.rtf in supplementary files for more details. Click here for additional data file. Copyright: © 2015 Morrell LJ and Joyce DA 2015 Data associated with the article are available under the terms of the Creative Commons Zero "No rights reserved" data waiver (CC0 1.0 Public domain dedication).

Device Users Responses to the questionnaire from students who used their own devices during the lectures
Please see Contents_of_data_files.rtf in supplementary files for more details. Click here for additional data file. Copyright: © 2015 Morrell LJ and Joyce DA 2015 Data associated with the article are available under the terms of the Creative Commons Zero "No rights reserved" data waiver (CC0 1.0 Public domain dedication).

Clicker Users Responses to the questionnaire from students who did not use their own devices during the lectures
Please see Contents_of_data_files.rtf in supplementary files for more details. Click here for additional data file. Copyright: © 2015 Morrell LJ and Joyce DA 2015 Data associated with the article are available under the terms of the Creative Commons Zero "No rights reserved" data waiver (CC0 1.0 Public domain dedication).

### Demographics

Seventy-eight students completed the questionnaire. Of these, 44 (56.4%) used their own device, while 34 (43.6%) used only clickers. Throughout, we refer to those that used their own devices as ‘device users’ and those that used the clickers we provided as ‘clicker users’ (device users used clickers in the clicker-only lectures). Age and gender of the students reflected the make-up of the cohort: 53.9% male, 43.6% female (two students did not answer), and 65 students (88.3%) were aged 18–23, with five (6.4%) aged between 24 and 29, five (6.4%) between 30 and 35 and three (3.9%) students aged 36 or over.

Almost every student owned at least one type of device that could potentially be used, with a large proportion owning multiple device types. Sixty-seven students (85.9%) owned a smartphone, with 76.9% owning a laptop, 32% owning a tablet, and 9% owning a netbook or ‘other’ technology (including iPods and non-smart mobile phones). While 21 students (26.9%) owned only one type of technology, the remainder owned more than one (2:42.3%, 3:26.9%), with two students declaring ownership of four types of devices that could potentially be used as part of an audience response system (a smartphone, laptop, netbook and tablet). One student did not possess any type of technology that could be used.

As clickers are used in other modules the students may have taken (notably a level 4 module Ecology and Evolution), students were asked if they had previous experience of using clickers in lectures: 67.9% (53 students) had, while 32.1% (25 students) had not.

Of the 44 students that used their own device, 37 (84.1%) used a smartphone and five (11.4%) used a tablet, with the remaining two students using either a laptop or other type of technology. In terms of operating system, 59.1% used iOS, 38.6% used Android, and one student (2.3%) used Windows (on a laptop). Of the 34 students who did not use their own device to participate, 23 (67.7%) already had a device that they would be willing to use, while eight (23.5%) did not have a device that they would be willing to use (although all owned at least one device that could potentially be used). The remaining three students (8.8%) did not currently possess a device, but were thinking of getting one. These three students included the one who did not currently own any types of technology and two possessing only a laptop.

### Student perceptions of lecture types

**Figure 1.**
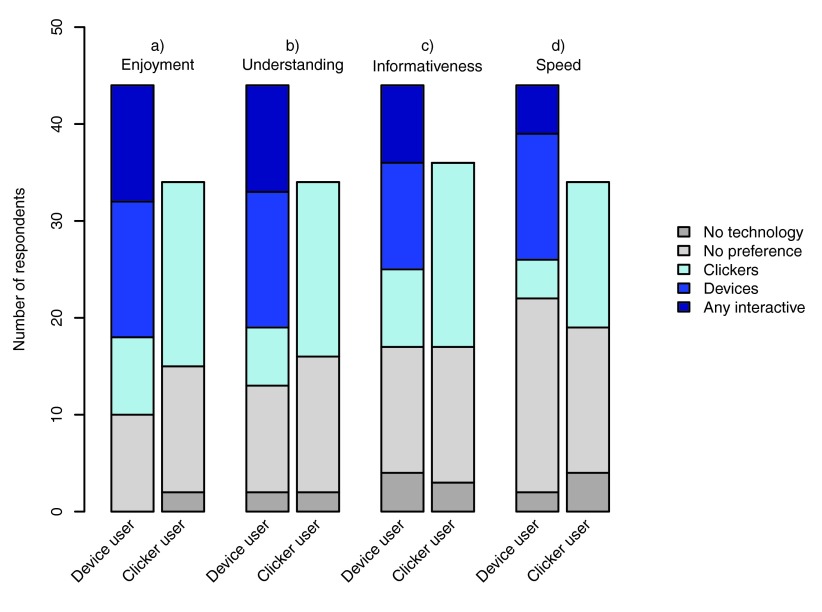
Student perceptions of the different types of lecture. Student perceptions of the three different types of lecture (no interactive technology, clicker and personal devices as response systems), in terms of their **a)** Enjoyment, **b)** Understanding, **c)** Informativeness and **d)** perception of the speed at which the lecture passes (fast = good), for students that used their own technology (device users) and those that used clickers (clicker users).

We asked students which type of interactivity was more enjoyable, informative, understandable, and made lectures seem to pass more quickly. Figure 1 summarises the responses. In general, device users felt that interactive lectures (either using their own devices or using clickers) enhanced their enjoyment and understanding slightly more than clicker users did, although these differences were not significant. Device users who preferred interactive lectures were divided in whether they preferred using clickers, their own devices or had no preference between the two types of technology (see Figure 1). These three preference categories have been grouped together as ‘interactive lectures’ in the analysis that follows. Overall, very few students felt the no-technology lectures scored most highly on any of the four measures.

In terms of enjoyment ( Figure 1a), 77.3% of device users (34 students) preferred lectures with interaction via either clickers or their own technology, compared to 55.9% of clicker users (proportion test: χ ^2^ = 3.107, df = 1, p = 0.078) preferring interactive lectures over the no-technology lectures. Significantly more than half of the device users preferred interactive lectures (binomial test: p < 0.001), but this was not true for clicker users (p = 0.608). None of the device users and only 5.9% of the clicker users (2 students) felt that the no-technology lectures were the most enjoyable ( Figure 1a).

For levels of understanding ( Figure 1b), 70.5% of device users (31 students) and 52.9% of the clicker users (18 students; χ ^2^ = -1.825, df = 1, p = 0.179) felt that the interactive lectures were more understandable. This represents significantly more than half of the device users (p = 0.010) but not the clicker users (p = 0.864). Two students (4.5%) who used their own technology and two students who used clickers (5.9%) felt that the no-technology lectures were more understandable ( Figure 1b).

Students expressed less clear overall preferences in terms of the informativeness of the lectures ( Figure 1c) or the speed at which they passed ( Figure 1d). 61.4% of device users (27 students) and 55.9% of clicker users (19 students; χ ^2^ = 0.598, df = 1, p = 0.439) felt that the interactive lectures were more informative than the non-interactive ones, but this was not significantly more than half (device users: p = 0.174, clicker users p = 0.608). Again, a few students (four device users and three clicker users) felt the no-technology lectures were the most informative ( Figure 1c). In terms of the speed at which the lecture appeared to pass ( Figure 1d), almost half the students (45.5% of device users and 44.1% of clicker users) had no preference with two device users and four clicker users feeling the no-technology lectures passed more quickly. The remaining 50% of device users (22 students) and 44.1% of clicker users (15 students) felt that the interactive technology lectures passed more quickly (proportion test: χ ^2^ = 0.083, df = 1, p = 0.774, binomial tests, device users: p = 1, clicker users: p = 0.608; Figure 1d).

### Distraction

When asked whether having their device ready to answer questions distracted them from the lecture, 36 of the device users (81.8%) answered in the negative, while seven (16.9%) said that it did. A mixed response was highlighted in the focus groups:

“ *It was easier to me like, ah, I’ll just reply to this text quickly*”
  “ *I think I’d be more tempted because it’s like well I could just say I’m doing it for this purpose [answering the questions] when really I wasn’t*”
  “ *I don’t think it* [having your device ready] *makes a difference at all*”

**Figure 2.**
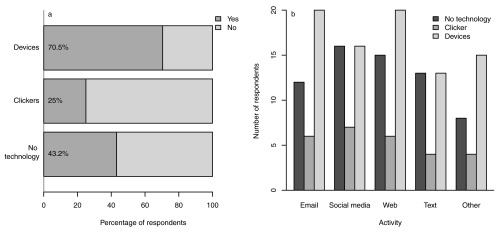
Use of personal devices for other purposes during lectures. **a)** Likelihood of device users using their device for other purposes during each of the three types of lecture (with no interactive technology, clickers or personal devices as responses systems). **b)** Number of students using their device for each broad category of activity (email, social media, browsing the web, sending or receiving text or other messages, and other activities) during each of the three types of lecture (with no interactive technology, clickers or personal devices as responses systems).

However, when asked which, if any of a number of web-based and interactive activities (including checking email, using social media, browsing the web and texting or sending a message) they carried out during each type of lecture, much higher numbers reported using their devices. Overall, 70.5% of device users admitted to using their devices for at least one other activity during the device lectures, compared to only 25% in clicker lectures and 43.2% in the no-technology lectures ( Figure 2a). Students were significantly more likely to self-report using their device for other purposes in the device lectures than the other two lecture types (proportion tests: device lectures vs clickers lectures, χ ^2^ = 16.443, df = 1, p < 0.001, device lectures vs no technology lectures, χ ^2^ = 5.604, df = 1, p = 0.018), but there was no difference in self-reported usage between clicker lectures and no technology lectures (χ ^2^ = 2.478, df = 1, p = 0.115). Thus, students are most likely to use their devices for other purposes when they are also using them as audience response devices.

Students used their devices for checking email, using social media, browsing the web, sending and receiving messages as well as other activities ( Figure 2b), with the majority of students reporting using their device for multiple purposes. When asked in the focus groups about the sort of activities they used their devices for during lectures, students in the first focus group suggested that it was mostly socialising rather than study-related, although the second focus group highlighted the academic activities that they might engage in, such as checking the Virtual Learning Environment (VLE) for assignments, going through the slides or using Google to search for information about unfamiliar topics. Both focus groups identified Facebook as being the primary “ *culprit*”. Other activities included online shopping for groceries and other items.

Twenty seven device users (61.4%) and 21 clicker users (61.8%) reported being aware of others using their devices for other purposes during a normal lecture. In the device lectures, this proportion increased slightly among clicker users (to 79.4%) but not device users (61.4%). When students in the focus groups were asked whether they noticed others around them using their devices for other purposes, students again answered affirmatively, but suggested that this was no more than usual. They did find that it could be distracting:

“ *I did notice a few people like on Facebook and things*”
  “ *I know a few times I’ve been in lectures where people next to me have been on a game and I’ll be like oh I’ll just go on that for a minute and then you kind of get into it and I’ve spent like a full 2 hours on a game*”
  “ *If they’re on the phone it’s not distracting. But if they’re talking then it will distract me*”
  “ *People are sometimes playing games and that’s distracting. You can see it out of the corner of your eye*.”

Interestingly, two students mentioned that they actively avoided taking their laptops and tablets to lectures because they find them much more distracting than their smartphones:

“ *I never take my laptop in… got an increased chance of getting distracted, because it’s there with a little button that says ‘internet’ on*”

However, despite admitting that they were more likely to get distracted, students felt that the interactive element of the lecture meant it held their attention more:

“ *I’m actually trying to think about the questions and actually answer it whereas before I’d just be sat there like*… [tails off]”
  “ *I was actually paying more attention to try and get the right* [answer]”
  “… *we actually had a discussion about the question, before we pressed it, so we were all a bit more attentive to the lecture*”

They also highlighted that factors other than having their devices available made them more likely to use them. Primarily, they suggested that they would be more likely to use their devices once they were already disengaged with the lecture:

“… *I’m getting bored and I have to go on my phone to keep awake*”
  “ *I don’t think I am distracted by it. I tend to turn to it when I’m bored or not engaged with lectures*”

They were also more likely to get distracted in environments (lecture theatres) that are perceived as being of lower quality:

“ *I’m more likely to use my phone in there*” [a lecture theatre identified by other students in the group as being “ *awful*” and having issues with echoes].

### Overall preferences

**Figure 3.**
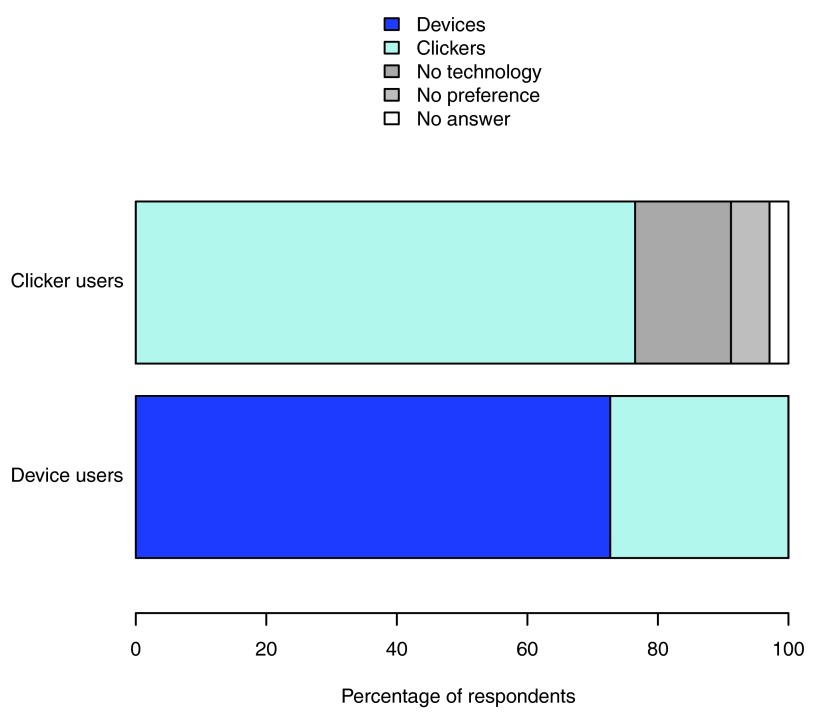
Preferences for different lecture types. The proportion of students who preferred lectures using their personal devices, clickers, and no interactive technology, by the type of response system they used in the device lectures (personal devices or clickers).

Of the 44 students who used their own device (device users), 39 answered the question “were you happy to use your own devices in this way?” with a positive response (88.6%) while five students (11.4%) would prefer not to, but would if it was the only option. No students responded “no” to this question. Of the device users 32 (72.7%; significantly more than half: p = 0.004) preferred the lectures using student-owned technology as response devices, while 12 (27.3%) preferred using clickers ( Figure 3). Of the 34 students who did not use their own devices (clicker users), 76.5% (26 students) preferred the clicker lectures (significantly more than half: p = 0.003), while 14.7% (five students) preferred the lectures with no interactive technology ( Figure 3; two students had no preference and one did not answer). No student who was unable to participate using their own device reported feeling disadvantaged in those lectures.

### Barriers to participation

Some other general themes emerged from the interviews and free text comments. Students mentioned that they would be unwilling to use their own devices if they had to pay for text messages or use up their data allowances, and highlighted software glitches as a potential concern for the wider use of the technology. Several students mentioned that battery usage would act as a barrier to their participation, particularly (as seemed to be the case with the software we used) if the device remained active rather than going into standby when the app was active, and required participants to log in again if the app was minimised. One student highlighted that their battery reserve decreased by 20–25 percentage points during a 1-hour lecture.

Although all the students who agreed to participate in the focus groups used their own devices, they identified a number of barriers to participation in their peers, including app availability (not available on Windows phones), the fact that those who did not have suitable technology would feel disadvantaged, particularly if compatible clickers were not available. One student suggested that this may lead to disengagement with the lecture:

“… *you put some people at a disadvantage if they don’t have a smartphone or whatever*…”
  “… *a few people said they didn’t feel involved in the lecture they just sit there and they’re like, well I can’t do anything so I’m just going to sit there, I’m not going to pay attention and then they’re probably more likely to go on their phones and actually like do something completely different just because like they couldn’t interact properly*”

Student participation is likely to vary widely depending on the software used, and as alternatives become available, some of the barriers highlighted here may disappear.

## Discussion

Our results reflect previous findings that students prefer interactive lectures using audience response systems ( Addison *et al.*, 2009; Keough, 2012; Sutherlin *et al.*, 2012). Both the students who used their own technology and students who used only clickers overwhelmingly preferred these lecture types to the traditional ‘hands-up’ or ‘shout-out’ interactive lectures, with the students using their own technology preferring this option to clickers ( Figure 3). Device users, in particular, were more likely to find these lectures enjoyable and felt that they increased their understanding ( Figure 1). Interactive lectures (and interactive teaching styles) are widely perceived as resulting in better student outcomes ( Freeman *et al.*, 2014) and thus the use of students’ own technology represents and effective way of overcoming some of the shortfalls associated with clickers, such as limited availability and the time associated with handing out and collecting them in ( Dunn *et al.*, 2013; King & Robinson, 2009).

We were particularly interested in whether having their devices ready on the desk to answer the interactive questions meant that students were more likely to use their devices for other purposes, thus becoming distracted from the lecture. Although 81.6% of device users reported that they were not distracted, 70.5% admitted that they used their device for a non-academic purpose during the lecture (particularly during the device lecture). This suggests that students did not equate using their devices for other purposes with being distracted from the lecture. The focus groups highlighted that in general, some students felt they used their devices for non-academic purposes when they were already distracted from (or bored by) their lectures. It is possible that, as highlighted by Sanbonmatsu *et al.* (2013), students who are multitasking by remaining engaged with the lecture and using their devices for non-academic purposes, do not feel distracted despite the fact that their cognitive performance maybe compromised. Further analysis using test scores and/or knowledge retention could examine this.

Previous work has shown that students use their phones or laptops ( McCoy, 2013) in class for a wide range of academic and non-academic activities that are outside the direct remit of the class, which may lead to reduced ability to remember lecture content ( Hembrooke & Gay, 2003). This has led to some calls for these technologies to be banned from the classroom, or for students to be banned from using them ( Shirky, 2014; Sørensen, 2014) including by the students themselves ( McCoy, 2013). Our results support the suggestion that encouraging students to use their technology for one purpose (e.g. as an audience response system) might also encourage them to use it for other purposes, and in this way, perhaps stand-alone clicker systems are more beneficial than using students’ own technology. Indeed, in this study, using a clicker system lead to a decrease in use of personal technology for non-academic purposes.

Although students may feel that they are able to multitask (simultaneously using their device for another purpose, yet not feeling they are distracted from the lecture), evidence suggests that people are not as good at multitasking as they think. In fact, individuals who most frequently multitask tend to be those who are least cognitively able to effectively carry out multiple tasks simultaneously ( Ophir, *et al.*, 2009; Sanbonmatsu *et al.*, 2013), and tend to be those who are less able to block out distractions and focus on a single task ( Sanbonmatsu *et al.*, 2013). This said, how much of an issue is it if students are distracted from lecture material? In the focus group interviews, several students suggested that being distracted in lectures was not necessarily a problem, as it is University policy that all lectures are placed on the VLE:

“ *If I’ve missed something… I can just go on eBridge* [VLE] *and go back through the slide and go through what I’ve missed*”
  “… *being distracted within the lecture isn’t so much of a deal if you can then go back to it and read it later and do wider reading.*”

Despite the increased distraction, there are a number of benefits to using web-based systems (outlined in the introduction), but awareness of the small number of students who do not possess the right technology, or do not want to use their own technology is necessary. Inclusivity in teaching is an important issue, and staff need to ensure that all students have access to appropriate technology that they are willing to use. The use of a system that allows the integration of apps and stand-alone clickers is one solution to this problem. Alternatively departments or Universities could provide compatible devices that could be used (e.g. iPods or tablets; Finkelstein *et al.*, 2013). Although the system we chose to test was cost-free to the students, another issue highlighted by the students was the use of battery life: opportunities for students to recharge their devices during the day are limited, particularly if they are moving between lecture theatres and classrooms. Widespread adoption of personal devices-as-clickers, such that students were using their devices in multiple classes each day, would exacerbate this issue.

Adopting personal technology to enhance student learning is an attractive prospect, in terms of both staff and student experience. However, the development of systems that minimise the barriers to use that we highlight is key. Additionally, an awareness of the potential for distraction is important and a discussion with students is recommended so that such distractions can be managed accordingly. Ultimately, interactive engagement during lectures enhances enjoyment and understanding and facilitating this method of engagement is valuable.

## Data availability

The data referenced by this article are under copyright with the following copyright statement: Copyright: © 2015 Morrell LJ and Joyce DA

Data associated with the article are available under the terms of the Creative Commons Zero "No rights reserved" data waiver (CC0 1.0 Public domain dedication). 

F1000Research: Dataset 1. Demographic data, 10.5256/f1000research.6207.d44001 ( Morrell & Joyce, 2015).

F1000Research: Dataset 2. Device Users, 10.5256/f1000research.6207.d44002 ( Morrell & Joyce, 2015).

F1000Research: Dataset 3. Clicker Users, 10.5256/f1000research.6207.d44003 ( Morrell & Joyce, 2015).

## Consent

All participants agreed to the use of their anonymised data (see ‘Morrell & Joyce – Consent and Questionnaire.pdf’ in the Supplementary Information).

## Supplementary information

Contents of data files & key to entries.

Consent Form and Questionnaire.

http://dx.doi.org/10.5256/f1000research.6207.s44005

Free text from questionnaires.

http://dx.doi.org/10.5256/f1000research.6207.s43985

Transcripts from semi-structured interviews.

http://dx.doi.org/10.5256/f1000research.6207.s43986
