# Supplementary material for: Interactive lectures: Clickers or personal devices?
Source: F1000Res. 2015 Mar 12;4:64. [Version 1] doi: 10.12688/f1000research.6207.1 (PMC4648207; doi:10.12688/f1000research.6207.1)
Supplement: Supplementary file 4 [file f1000research-4-6656-s0003.tgz › 81807e67-c6b0-454e-8b09-193d728c27f0.rtf]

Interactive lectures: Clickers or personal devices?
Lesley J. Morrell & Domino A. Joyce
Contents of data files & key to entries

Morrell&Joyce – Data – Demographics
This file contains the demographic details for all students completing the questionnaire. Please refer to the questionnaire (Supplementary Information) for details of the questions and possible responses.
For questions 6 &7, a Y indicates that the student ticked that box on the questionnaire, while a 0 indicates that they did not. For questions 8 and 10, responses are indicated by Y(es) or N(o). 

Morrell&Joyce – Data – Device users
This file contains the responses to section B of the questionnaire, for students who used their own devices in the lectures. Please refer to the questionnaire (Supplementary Information) for details of the questions and possible responses.
For questions 11 and 12, a Y in the relevant section indicates that the student ticked that box, while a 0 indicates that they did not. For questions 20 - 24, responses are indicated by Y(es) or N(o), or 0 for no response to that particular question.
For the questions 13 and 14, the following coding has been used:
Question 13: Were you happy to use your devices in this way?
Y – Yes
P – I'd prefer not to, but I will if I have to
N - No
Question 14: How comfortable did you feel in each of the lecture types?
1 – Very comfortable
2 – Quite comfortable
3 – Neutral
4 – A bit uncomfortable
5 – Very comfortable
Responses to questions 19 and 23 have been excluded from this data sheet as they were not used in the analysis due to issues with the way these questions were completed by the students in relation to their previous answers.

Morrell&Joyce – Data – Clicker users
This file contains the responses to section B of the questionnaire, for students who did not use their own devices in the lectures. Please refer to the questionnaire (Supplementary Information) for details of the questions and possible responses.
For questions 28, a Y in the relevant section indicates that the student ticked that box, while a 0 indicates that they did not. For questions 26, 27 & 30, responses are indicated by Y(es) or N(o), or 0 for no response to that particular question.
For question 25, the following coding has been used:
Question 25: Do you have a personal device that you might be willing to use:
	Y – Yes
	Maybe – No, but thinking of getting one
	N – No
Responses to question 31 have been excluded from this data sheet as they were not used in the analysis due to issues with the way these questions were completed by the students in relation to their previous answers.


Free text comments from the questionnaires can be found in Morrell&Joyce – Free Text Comments.pdf
