# Supplementary material for: Interactive lectures: Clickers or personal devices?
Source: F1000Res. 2015 Mar 12;4:64. [Version 1] doi: 10.12688/f1000research.6207.1 (PMC4648207; doi:10.12688/f1000research.6207.1)
Supplement: Supplementary file 5 [file f1000research-4-6656-s0004.tgz › b4afbfee-1514-403f-aa8e-6cf151805272.pdf]

UNIVERSITY OF Hull

Testing personal devices as interactive audience response systems

As part of our continuing efforts to improve provision in the Biosciences, and better understand the learning motivations, experience and preferences of our students we would like to thank you for taking the time to complete this survey and agreeing to take part in our project.

The information collected through this questionnaire will be anonymous and only used by us (your lecturers) in relation to our work to understand the value of personal electronic devices (smartphones, tablets and laptops) in lectures. In this context it is possible that the data generated will be presented to the wider learning community through conference presentations and published journal articles.

We may also wish to carry out some follow-up work (surveys and interviews) with you based on some of your answers to this questionnaire. If you would be happy to take part in our future research it will be important for us to be able to link your responses to this survey to those of future surveys. This of course can only be achieved if you agree to our knowing who you are, so please add your contact details when prompted. Undergraduate interns will collate the responses from these forms and carry out interviews and follow up work, so your lecturers will not be able to associate comments with particular students.

Data generated by the project will be stored electronically for analysis beyond the end date of the funded project, but it will only be used for the purposes outlined i.e. to understand the value of personal electronic devices in lectures.

Dr Lesley Morrell, Dr Domino Joyce

Please return this questionnaire as you leave, or to the School reception.

By completing this survey, you agree that:
I understand that:
1. Upon receipt, my questionnaire/interview will be coded and my name and contact details kept separately.
2. Any information that I provide **will not** be made public in any form that could reveal my identity to an outside party (i.e. that I will remain fully anonymous).
3. Aggregated results will be used for research purposes and may be reported in scientific and academic journals.
4. Individual results **will not** be released to any person except at my request and on my authorisation.
5. That I am free to withdraw my consent at any time during the study, in which event my participation in the research study will immediately cease and any information obtained from me will not be used.

In addition, on completion of the module, we may wish to access your marks. If you are willing for us to do this, please tick the box when asked.

A: Relevant information about you

1. Student number _____________ Tick if you are willing to be contacted ☐

Email address for contact ____

2. Are willing to allow us to access your marks on completion of the module?

Yes ☐ No ☐

3. Age at 1 January 2013: ____ years old

4. Gender: Male ☐ Female ☐

5. Programme of Study: ______________________________

6. Which of the following types of technology do you own? Tick all that apply

Smartphone ☐

Tablet ☐

Netbook ☐

Laptop computer ☐

Other portable device (please specify)_____________

7. In each of the following modules, there were lectures where no interactive technology was used, lectures using clickers handed out by the lecturers, and lectures where students were asked to use their own devices to respond to the lecturers questions (smartphones/tablets/laptops). Please tick the following table to indicate which lectures in which modules you attended, focusing only on the lectures in the module given by the lecturers named below:

	No interactive technology	Clickers handed out by the lecturer	Students using own device
Behavioural Ecology (Dr Lesley Morrell)	☐	☐	☐
Evolutionary Biology (Dr Domino Joyce)	☐	☐	☐
Cancer Biology (Dr Stephen Maher)	☐	☐	☐

8. Prior to this academic year, have you used interactive technology in a lecture?

Yes ☐ No ☐

9. How digitally literate to you consider yourself relative to others on your course?

More literate ☐ About the same ☐ Less literate ☐

B: Using your own interactive technology in lectures

Thinking **ONLY** about the lecture where students were using their own device to answer questions:

10. Did you use your own device to respond to the lecturers' questions?

Yes ☐ (continue from **question 11**) No ☐ (go to **question 25**)

Only answer questions 11-26 if you used your own device in lectures

11. Which of the following did you use?

Smartphone ☐

Tablet ☐

Netbook ☐

Laptop ☐

Other ☐

12. Which operating system does the device you used have (please specify the version if you can)?

Android ☐ ________________

iOS /Mac OS ☐ ________________

Windows ☐ ________________

Linux ☐ ________________

Other (specify) ☐ ___________________________

13. Were you happy to use your own devices in this way?

Yes ☐

I'd prefer not to, but I will if I have to ☐

No ☐

14. How comfortable did you feel in each of the lecture types (tick as appropriate)?

	Very comfortable	Quite comfortable	Neutral	A bit uncomfortable	Very uncomfortable
No technology	☐	☐	☐	☐	☐
Clickers	☐	☐	☐	☐	☐
Own devices	☐	☐	☐	☐	☐

15. Overall, which interactive approach did you prefer?

- Clickers handed out by the lecturer ☐
- Using my own technology ☐
- I preferred the lectures with no interactive technology ☐

16. Which system made the lectures more (tick as appropriate):

	No technology	Clickers	Own devices	No preference
Enjoyable	☐	☐	☐	☐
Informative	☐	☐	☐	☐
Understandable	☐	☐	☐	☐
Appear to pass more quickly	☐	☐	☐	☐

17. Did you do any of the following in the lectures (please tick all the apply, for all types of lecture):

	No technology	Clickers	Own devices
Check email	☐	☐	☐
Use social media for academic purposes	☐	☐	☐
Use social media for non-academic purposes	☐	☐	☐
Browse the web for academic purposes	☐	☐	☐
Browse the web for non-academic purposes	☐	☐	☐
Text (or otherwise send a message e.g. BBM, DM)	☐	☐	☐
Used my device for other purposes	☐	☐	☐
I didn't use my device apart from when asked to	☐	☐	☐

18. If you used your device for other purposes, please specify broadly the type of use (e.g. used word to take lecture notes, used word to work on coursework)

19: If you used your device, how much time (approximately) did you spend on activities other than responding to the lecturers' questions (i.e. those you identified in question 17)?

	No technology	Clickers	Own device
Less than 2 minutes	☐	☐	☐
2-5 minutes	☐	☐	☐
5-10 minutes	☐	☐	☐
10-30 minutes	☐	☐	☐
Over 30 minutes	☐	☐	☐

20: Do you feel that having your device ready to answer questions distracted you from the lecture?

Yes ☐ No ☐

21: If you did not use your device for other purposes, were you tempted to?

Yes ☐ No ☐

22: Were you aware of others around you using their devices for other purposes?

a) The lectures where personal devices were used as response systems

Yes ☐ No ☐

b) The lectures where personal devices were NOT used as response systems

Yes ☐ No ☐

23: Thinking about the person you saw using their device the most, how much time did they spend using their devices for other purposes?

	Own device lecture	All other lectures
Less than 2 minutes	☐	☐
2-5 minutes	☐	☐
5-10 minutes	☐	☐
10-30 minutes	☐	☐
Over 30 minutes	☐	☐

24: Did other people using their devices for other purposes distract you from the lecture?

Yes ☐ No ☐

Please go to question 32. The following questions are for students who did not use their own devices in the lectures

Only answer questions 25-31 if you did NOT use your own device in lectures:

25. Do you have a personal device that you might be willing to use in this way?

Yes ☐ No, but thinking of getting one ☐ No ☐

26. If you did not use your own technology, did you use the clickers provided in the smartphone lecture?

Yes ☐ No ☐

27. Did you feel disadvantaged because you didn't have a smartphone / tablet etc?

Yes ☐ No ☐

28. Which system made the lectures more (tick as appropriate):

	No technology	Clickers	No preference
Enjoyable	☐	☐	☐
Informative	☐	☐	☐
Understandable	☐	☐	☐
Appear to pass more quickly	☐	☐	☐

29. Overall, which lecture approach did you prefer?

Clickers handed out by the lecturer ☐

I preferred the lectures with no interactive technology ☐

30: Where you aware of others around you using their devices during:

a) The lectures where personal devices were used as response systems

Yes ☐ No ☐

b) The lectures where personal devices were NOT used as response systems

Yes ☐ No ☐

31: For each type of lecture, think about one person who used their device. How much time did they spend using it? (leave blank if you were not aware of others using their devices)

	Personal device lecture	All other lectures
Less than 2 minutes	☐	☐
2-5 minutes	☐	☐
5-10 minutes	☐	☐
10-30 minutes	☐	☐
Over 30 minutes	☐	☐

This question is for all students

32. Are there any other comments you would like to make about the use of interactive technology in lectures?

Thank you for completing this survey.
