# Supplementary material for: Interactive lectures: Clickers or personal devices?
Source: F1000Res. 2015 Mar 12;4:64. [Version 1] doi: 10.12688/f1000research.6207.1 (PMC4648207; doi:10.12688/f1000research.6207.1)
Supplement: Supplementary file 6 [file f1000research-4-6656-s0005.tgz › 4f1ec94a-1337-484f-bf0b-769ecda66942.rtf]

Interactive lectures: Clickers or personal devices?
Lesley J. Morrell & Domino A. Joyce
Free-text comments from questionnaires

At the end of the questionnaire, students were given the opportunity to respond to the free-text question “Are there any other comments you would like to make about the use of interactive technology in lectures?”. These free-text responses are printed in full below, divided into students who did not use their own device (table 1) and those that did (table 2). Participant numbers relate to those found in the data spreadsheets..

Table 1: Free-text comments from the questionnaires from students who did not use their own devices, but used clickers in both 'clicker lectures' and 'device lectures'.
Participant	Comment	
1	I don't feel it makes a huge difference despite it passing time. Also, it was interesting to see how everyone else in the lectures answered the questions, but again, I don't believe it makes any significant impact on my learning	
13	I find it enjoyable	
14	I enjoy using the clickers and I think using personal technology is a good move forward. However I do own a smartphone but as it is a Windows phone I was unable to partake in the lecture where they were using. Looking in to an app for this would be good. I also intended to use my laptop to participate but I couldn't get it to work.	
17	Good to use a few times in a lecture, but try not to overdo it.	
18	A nice idea	
23	It seemed easier for people with iPhones to set up. I have Windows phone and couldn't get it to work. Some people complained it flattened their battery quicker.	
27	Makes the lectures more fun. Less likely to zone out.	
29	The use of technology in the lectures makes them more interesting and gives a motive for students to actually listen and not start playing with their phone, sleep etc. I think that there should be some kids of point system for each individual to give a motive for students to actually try and answer than clicking a random button.	
31	Wasted more time than is needed as we are all busy at the moment, so all this is redundant and we could just be told what it is, rather than waste time like this in the lecture as we didn't finish it.	
37	Make sure that the interactive technology i.e. clickers work all the time, as sometimes they did not seem to register properly on the system so my choices were not picked up.	
44	It is good as a method of checking understanding throughout lecture.	
45	Helps consolidate understanding of a topic as it makes you think and apply your knowledge during the lecture, rather than just listening to someone talk for an hour or two.	
46	Really good, further pushes a point, breaks up lecture.	
48	I think they are a positive as they increase the level of interaction and break up the lecture a bit.	
51	People are more willing to answer – anonymity. Becomes rewarding to be 'right' – game like. Learnt more when I was wrong. Due to dedicated time for question and answer – time to consider my answer during downtime between explanation of slides.	
55	I like the clickers but it didn't massively affect my learning and enjoyment of the lecture. I think just sticking with the clickers would be a good idea. I struggle to see the point in going to all these lengths to make apps etc. when there was nothing wrong with the old clickers anyway.	
57	Good to make sure people are paying attention in lectures. Not great if used too much (wastes time answering questions which we haven't been taught about yet).	
59	Good use of videos makes the lectures more engaging.	
61	Makes answering questions anonymous so beneficial and increases understanding of lecturer as you have to think about an answer.	
63	Interactive technology can be a way forward to learning however we need to consider the amount of power used, leading to drained batteries. I prefer the clickers as it is more simple to use and don't need to set up the system on tablet, iPhone, smartphone etc.	
68	I had to use a clicker even though I had a smartphone as there wasn't an app made available for my smartphone's operating system (Windows phone).	
70	I feel they are a waste. The computer always froze taking up class time and causing the lecturer to rush. I also don't see the purpose of them. Ask the question then explain the correct answer.	
74	If it doesn't shut down as much it is a very helpful technique. I think I pay attention more with questions thrown in.	
75	It would be better if everyone used the clicker devices that way mobile phones are not used.	
77	It's better than being talked at for an hour.	
78	I enjoy the lectures where it is used. Koolio.	

Table 2: Free-text comments from the questionnaires from students who used their own devices in the 'device lectures' and clickers in the 'clicker lectures'.
Participant	Comment	
2	I do not like to use my smartphone as a clicker as it drains my battery quickly and so I may be left without a phone until I get home to charge it.	
6	It drained my phone battery	
21	No real preference between clickers and own device. Although with own device spent less time using phone for non-academic purposes.	
24	Using own devices drained the battery significantly. However, it made lectures more enjoyable and interactive.	
30	Good idea!	
36	Breaks lecture up. More enjoyable. Reinforces learning. Ensures the lecture is not just a repetition of the PowerPoint read verbatim by the lecturer.	
38	Kept the lecture interesting, as there was some form of interaction rather than the lecturer talking at you. The questions, I found, made me pay attention more on the whole in order to answer them.	
42	I preferred using my own device although I was more inclined to go on Facebook/Twitter.	
43	I found that using my smartphone made me more likely to get distracted due to notifications.	
47	Don't mind using phone or clickers. Enjoy interactive lectures more than normal.	
50	Lecturers need to explain the answer after we have answered the question. Often this is forgotten.	
56	I think it is really helpful and keeps attention throughout the lecture.	
58	I think it was a good way of keeping it interactive and interesting.	
62	I liked using interactive technology, I feel more involved in the lecture. I learn better when being interactive. Using the technology for other things I think would be fun and makes it easier to learn the subjects, as you're more involved and not just listening to the lecturer.	
64	I do not understand why I have to type in my student ID whilst using vPad, as it's off-putting. Since I am not being assessed on my vPad answers, I think the answers I provide should be done anonymously.	
65	Using student ID is off-putting – I'd rather you didn't know what answers I put as it's not an exam. It should be anonymous.	
66	People use phones regardless of if they are included in the lecture. Being on vPad makes it harder to go on something else therefore probably better on own device.	
67	The interactive lectures offer a happier learning environment to me as I can see an instant result to the questions asked. The iPhone app rapidly drains batter as it leaves the screen on all the time.	
76	The software often crashed, losing lecture time. This should be fixed.	
