# Supplementary material for: Interactive lectures: Clickers or personal devices?
Source: F1000Res. 2015 Mar 12;4:64. [Version 1] doi: 10.12688/f1000research.6207.1 (PMC4648207; doi:10.12688/f1000research.6207.1)
Supplement: Supplementary file 7 [file f1000research-4-6656-s0006.tgz › 20402cff-f4b3-4163-9de3-862786997d97.rtf]

Interactive lectures: Clickers or personal devices?
Lesley J. Morrell & Domino A. Joyce
Transcripts from focus group interviews

Names of individual staff identified by the participants have been removed and replaced with [lecturer]. 
Text is as transcribed by Khia Dobbinson and Matthew Walker (interview 1) and Julie Furnell (interview 2).

Interview 1: May 2014
Participants: Interviewer 1 (I1), Interviewer 2 (I2), Participant 1 (P1) and Participant 2 (P2).

Interviewer 1: So I guess the first thing is how did you feel about using -- you both used your phones didn't you?
Participant 1: Yeah.
Participant 2: Yeah, I did.
Interviewer 1: So were you both comfortable using your phones?
Participant 2: Yes.
Participant 1: I preferred using my phone.
Interviewer 2: You preferred that to using a clicker?
Participant 1: [nods in agreement]
Participant 2: Yeah so did I.
Interviewer 2: Is that just because you were more comfortable using your phone or --.
Participant 2: Yeah yeah, yeah I kept doubting whether I'd done the right answers on the clicker [laughs].
Interviewer 2: Right ok so the familiarity with your own device helped. Did you find then, with your phones, that you were getting maybe more distracted than you would or --?
Participant 1: I did a little bit yeah.
Interviewer 2: You did a little bit?
P1: Yeah.
I1: Is that because your phone was already out?
P1: Yeah.
I1: So it was a little bit, rather than --.
P1: It being in my pocket. It was easier to be like ah I'll just reply to this text quickly.
P2: Yeah same. Yeah, easily more distracted. 
I1: Yeah? Do you think then having permission then to have you phone out in the lecture meant that you were maybe more likely to use it for other things like that or would it be equally as distracting if it was just in your pocket?
P1: It's probably be equally, but I think I'd be more tempted because it's like well I could just say I'm doing it for this purpose when really I wasn't.
I2: Right, OK.
P2: I'd have used it more -- I'd probably used it the same amount whether it was out or in my pocket anyway.

I1: What about other people around you, was that distracting? And -- so if everyone was using their phone to check their email or quickly reply to texts did you notice that?
P1: I did notice a few people like on Facebook and things.
I1: More than usual?
P1: No not really, about the same.
P2: Yeah probably about the same.
I2: If you could -- because this might be quite difficult -- if you could think of the time that you spend on the internet during lectures, how much do you think is work based and how much is socialising, attending to personal things and things like that?
P1: Mine's a lot more socialising.
I2: Yeah? Could you put it at a percentage do you think or --?
P1: Probably about 80% of the time.
I2: About 80% of … being social.
P2: I'd say mine's probably about 70.
I2: OK. So you do both use the internet on your phones during lectures for things that are --.
P1: Yeah.
I2: But it is more social.
P2: I'm a keen Googler.
I2: You're a keen Googler? So what sort of things would you Google?
P2: If there's something that looks interesting on the slide that maybe they've not gone into I'll normally Google that [laughs] and do a little bit of further reading mid lecture, sort of thing.
I2: OK. Does that distract you from what's happening or --?
P2: It does, but then I always feel like if it's on the slide I'll always read it back later when I go through the slides anyway. 
I2: Yeah. 
P2: I'm not going to miss the lecturer reading the title of the next slide. 
I2: OK. So you think you maybe benefit from that then?
P2: Yeah.
I1: Does that depend on what type of -- who it is lecturing, because I know for instance with [lecturer] at the bottom of all her slides she has more or less what she says.
P2: Yeah, yeah. With her she basically has a script of what she talks about so I always know -- even if I do miss something important, I always know at the bottom of her lectures they're there or if someone else that I know has recorded the lectures I can always ask them for a copy of the recording. 
I2: OK. Would you say that work stuff then things like Googling mid lecture or social stuff, which one would you find more distracting from what's going on? 
P2: I'd say easily Facebook.
P1: [nods in agreement].
I2: Facebook, Facebook's the main culprit.
P1: Yeah.
Interviewer 2: Do you know how to turn of notifications?
P1 & P2 [simultaneously]: Yes.
I2: But would you?
P1: I do but then I still go and check.
I2: You would still check anyway even if you weren't getting direct notifications?
P1: Yeah.
P2: I get them and ignore them half time.
I2: OK.
P2: Just delete them so -- if they don't seem relevant if or they're not important to me, I'll still ignore it sometimes.
I1: So sort of coming back a little bit to the point that you just made about looking stuff up in lectures do you find it useful in that -- from that point of view, you know, looking up new stuff or related things in terms of does it help your learning a lot?
P1: I do that if I've missed something, like if they've gone a bit quick from -- going through I can easier just go on eBridge [VLE] and go back through the slide and go through what I've missed and I find that quite useful.
P2: Yeah I agree.
I2: So what devices do you usually use in lectures? Do you take in laptops and things or do you just have your phones --?
P2: I never take my laptop in --.
I2: You never take your laptop in?
P2: No. I find that's even more -- got an increased chance of getting distracted, because it's there with a little button that says Internet on.
I2: [laughs].
P2: Oh I've got a direct link to Facebook!
Everyone laughs
P2: No I take either my phone or my Kindle.
I2: OK, so you have your Kindle as well.
P1: I used to take my iPad but I got distracted.
I2: OK, so you both actually actively avoid taking in [laughs] technology that is too distracting.
Everyone laughs
P1: Yeah.
I2: OK. Do you think -- you know quite a lot of lecturers might not like you using your phone in a lecture, do you think there's a way that they could stop people from using their phones?
P1: I don't think they really could unless they like deliberately say it but I mean I've got caught using my phone and a few times someone's complained and said well if she wants to pay for her tuition fees and sit there playing Candy Crush, it's her own fault.
I2: Yeah.
P1: But I mean because I know quite a few people who sit there and just play games, sometimes I find like, in some of my lectures, they'll deliberately walk by and like just stand there for a bit so you kind of get paranoid like they're looking at me I'm going to put it away now.
I2: OK.
P1: But I don't know whether they could just officially just stop it because it's quite easy.
I2: Yeah, yeah.
P2: I don't. They only way they'd stop it is taking people's phones off them. It's like you say with the tuition fees, we're at the age when we come from University it's our personal choice now. If we don't want to learn, no matter what the lecturer says it's not going to change it.
P1: Plus you can't prove exactly what they're using it for, they could actually be using it to, for, like on eBridge and actually going through it so it would be hard.
Everyone laughs
I1: So is that sort of stuff increasing there's now Wi-Fi immersed lecture theatres? 
P1: Yeah.
I1: I'm tempted to say all but I … --.
P1: Yeah.
I1: Is that definitely something -- because I'm guessing in first year that it probably wasn't in most of them. 
P1: No, a few of them it was quite hard to try and get it. 
I1: OK.
I2: So just going back to -- you said you stopped taking in your iPad and you don't take in your laptop because you find them too distracting, do you think using the internet in general then is quite distracting?
P1: It can be yeah because it's quite easy to just easily turn over to another tab and then just be like oh I'll just look at this quickly and then you kind of just get carried away.
I2: So do you think that matters then in your lectures, if you're distracted or not?
P1: I think it can be like because I know a few times I've been in lectures where people next to me have been on a game and I'll be like oh I'll just go on that for a minute and then you kind of get into it and I've spent like a full 2 hours on a game
P2: [laughs].
I2: So it sounds like you do get a bit distracted by people around you as well.
P1: Yeah.
I2 [addressing P2]: Whereas you weren't so much?
P2: No, I don't normally.
I2 [addressing P1): But you do?
P1: I do, yeah.
I2: They inspire you, to waste time [laughs].
Everyone laughs
I2: Can you multitask, do you think? For example, could you Google something and still be keeping half an ear on what's going on in the lecture, or if you are distracted by the internet are you completely engrossed in that?
P1: Yeah I will be, I will completely miss whole lectures and just be like – I then go on revision and be like, I've never even heard of this.
I2: [laughs] oops.
P1: But yeah I do tend to get like very distracted and it is -- I find it easier if I'm taking notes and just writing out everything, that's when I take it in but if I'm just sat there and I'm not really paying attention, I won't take any of it in at all.
I1: So having either the clickers or using your phone to participate in this sort of interactive thing does that hold your attention a little bit more?
P1: Yeah it does with me because I'm actually trying to think about the questions and actually answer it whereas before I'd just be sat there like … . 
P2: Yeah.
I2: So do you think you were less likely to get distracted from the lecture content when you had this interactive element?
P1 & P2 [simultaneously]: Yeah.
I2: Using clickers or phones to answer questions? 
P1 & P2 [simultaneously]: Yeah.
P1: because I'd also be kind of thinking about if somebody could see, because I know a few people like look at what you're pressing to see if you go the right answers so if they find out you've pressed the wrong button id get a bit worried about like are they going to like laugh because I've got the complete wrong one --.
Everybody laughs
P1: And it was so obvious and so I was actually paying more attention to try and make sure I got the right one.
P2: Yeah.
I2: OK. So do you think then that -- well do you think that using them had a positive effect on your learning during the lectures?
P1 & P2 [simultaneously]: Yes
P2: Definitely, definitely. I know that in one of the larger lecture theatres we were sat in a row with some of my friends and we actually had a discussion about the question, before we pressed it, so we all kind of were a bit more attentive to the lecture.
I2: Right OK so you were really engaged.
Interviewer 2 asks Interviewer 1 to check the interview is still being recorded.
I1: OK so now I'm assuming you've come to the point where you're starting to revise
P2: Yes.
P1: [looks worried] ish [laughs].
I2: Don't worry! [laughs].
P1: I've been busy with all my other assignments!
I1: It's perfectly fine if you haven't. So if -- in the case of which you have started revision, are the things from these lectures coming back a little bit more clearly because you were paying more attention or -- compared to those lectures that weren't interactive?
P2: Yes. Yeah definitely. Especially for my lectures I can actually remember like what some of the questions were and my answers to them, I think because I was a bit more involved in the lectures instead of just sat there listening to Lesley talk she was like, right, this is on about bird foraging and gave us like some graphs and we had to choose the correct graph and I can remember which graph was right so it's kind of made me remember better.  
I1 & I2 [simultaneously]: Yeah?
I1: That's good. 
P1: and I kind of picked up on more on which bits I didn't know so I could go now and revise the bits that I didn't know and it's helped like not just identify what I did know but what I didn't know.
I1: Which is always useful because you need to know where to start looking at stuff.
I2: Do you think if – so obviously this was quite novel wasn't it? It's not something you do in all of your lectures. Do you think if you were doing it every lecture you had with [lecturer] or, you know, all of lectures that you had with anyone, do you think it would still be as effective?
P2: I think the novelty would wear off after a while but I still think it would probably be quite beneficial in the long run.
P1: Yeah I think it would because I think most of the lecturers are just them talking to you and you end up getting that bored that -- I mean I know for me quite a lot of my lectures and they're just talking and I'm getting bored I have to go on my phone to try and keep awake so I'll just be like, I can't -- I don't have a clue what you're talking about and I'm bored and I'm like actually been falling asleep in a lectures and have been like right I need something to distract me.
I2: So you think that anything that would try to engage you more would have a positive effect even if it wasn't just a novelty?
P1: Yeah.
P2: I quite liked the clickers because they weren't asking you personally asking you for your answers so I wasn't personally worried if I got the question wrong because like it's just a number on the board but when the lecturers like 'right guys, you!' and asks a questions I get like no, don't look at me, don't talk to me, I don't know if I know the answer
I1: I was just going to ask what sort of the difference between the lecturers just asking a question and having some sort of interactive and is it that sort of anonymity that's appealing?
P1 & P2 [simultaneously]: Yeah
P2: I hate the lecturer when the start, like as soon as they start asking questions to the room I'm like straight down in my seat, like as small as possible.
P1: That's what I'm like but I also think It helps like get the interaction but it's not just like, it makes it a bit more like informal kind of thing, you can actually have a discussion with other people around you rather than just sat there and being like really silent, it's quite a good way to make new friends kind of thing.
I2: That's a good point, that's not something I've thought of, so basically it's encouraging discussion. 
P1 & P2 [simultaneously]: Yes.
I2: A bit of back and forth with you and lecturer but also people in your group as well.
P1: Yeah.
I1: Have you had any lectures where they've allowed time for discussion? You know, just normal lecturers where they've said have a think about this or --.
P1: A few times yeah they've actually, you know, talk to people in your group about what you think about this and then like you go and share what you've discussed and I think it's good because then you're not worrying about oh what do I say at the start it's just, it's a good what to actually talk to people.
I1: And it's that same thing with keeping you engaged as well.
P2: Yeah, I've had quite a few lectures where they've put us into groups and made us talk about stuff.
I1: That's good, because I know in a lot of my lectures you didn't really get the opportunity to discuss and then on the odd occasion that you did everybody was that used to being quiet [laughs] that you didn't have any discussion really anyway. 
P1: Exactly, you just sit there.
I1: Yeah kind of silent, not really knowing.
P1: Or we tend to discuss other things apart from work because we didn't know what else to talk about.
I2: You both -- earlier on, you both said you preferred using your phones to clickers, would you – would you mind -- would you be willing to potentially pay or use up minutes and things like that or use up internet allowances if say, Wi-Fi wasn't available?
P2: I don't mind using my texts, I've had where one of my lecturers has made us text in --.
I2: OK.
P2: Text in answers before, and that didn't bother me because like more people get loads of minutes but for the Internet basis I probably wouldn't be willing.
I2: You wouldn't want it to eat up that?
P2: No I'd probably just turn my Internet off.
P1: Yeah. It depends on how much Internet usage I've got, if I've got quite a lot then and it doesn't use much then I wouldn't mind but if it uses quite a lot then I'd be kind of like I'd rather save it. 
P2: Beginning of the month sort of thing then I'd use it, yeah [laughs].
I2: And what about texts for you [P1]?
P1: Well I have unlimited texts so I don't mind texting.
I2: So you don't mind, you don't mind that? 
P1: No.
I2: Can you think of anything that would make you less willing to use you phones in this way?
P2: Well if I couldn't get a signal or anything then I'd kind of like -- well I'm not going to use it because obviously you can't. I don't know, I think I would probably prefer to use my phone.
I2: You would? And did either of you have any problems with the app or anything like that because that was something we saw sometimes [to I1] wasn't it?
I1: Yeah some people had issues installing it.
P1: Yeah well sometimes like with mine when I did it if you came off that screen it'd make you re-log back in and you had to wait a bit for it to synch back up which was a bit annoying so you'd have to keep like making sure your phone was still lit up which could use your battery up.
I2: Right OK.
P2: Yeah it was a bit draining. I had issues at the beginning due to my own technological idiocy, it took me a while to log in but I think that because the app wasn't very clear, I didn't know what numbers I was typing in where, which password was what, sort of thing.
P2: How were you with battery usage and when you'd come to the end of this was that an issue or --.
P2: Yes.
P1: Yeah.
P2: It was?
P1: Yes.
P2: I think in an hour lecture it went down by 20, 25%. 
I2: So you think maybe then if you had, if you had maybe two or three lectures in a day that were using the same sort of method then it would be less feasible?
P1: Yeah it would.
P2: Which is where the clickers were the better alternative obviously. 
I2: Yeah, so they were better for that?
P1 & P2 [simultaneously]: Yeah.
P1: But the clickers were a bit annoying because sometimes they wouldn't register your votes or like in a few lectures they wouldn't just, the program wouldn't work with clickers it'd only work with app ones.
I1: OK. So if there was problems specifically with the app either due to the instructions not being quite clear or other things would it be preferable to have some sort of internet based system instead of an actual like, an app that you had to log into it went to a website and logged in and did it that way?
P1: Yeah I think probably would be like, at least have the back up so that you know that it would work if you can't get the app. 
I1: OK, because from I kind of understand from what's vaguely been mentioned, you know, there's quite a few different systems out there that all do a similar sort of thing, so it's interesting to know if that would be --.
P1: Yeah because I think some phones, like the newer phones -- like I think Windows can't get quite a lot of apps, so if you've got a particular phone you might not be able to get the app and it might put you at a disadvantage compared to others.
P2: Yeah.
P1: Did you know -- did any of your friends have any problems with it or --?
P1: I think a few of my friends have got Windows phones so they couldn't download it so they did have to use the clickers and they were a bit annoyed because everyone else was using their phones.
I2: OK.
P1: So they did feel a bit like, left out about it.
I2: Right OK.
I1: So I know that you obviously can't answer for you friend but, because you were both using your phones and were reasonably happy with it, what about those people if the clickers weren't available and they couldn't have used their phones, how do you think they would have -- ?
P1: I think that quite a few of them would have felt kind of left out and like they'd be kind of, I know a few people that said that they didn't feel involved in the lecture they just sit there and they're like, well I can't do anything so I'm just going to sit there, I'm not going to pay attention and then they're probably more likely to go on their phones and actually like do something completely different just because like they couldn't interact properly. 
I1: So in the lectures was it sort of more or less compulsory that you took part? Did you feel that, you know, you had to, you really had to take part in this?
P1: No, like well because obviously it just came up with a number of how many was in the group, like it would be noticeable if like quite a large amount didn't but if like the odd two didn't it didn't really make much difference because they wouldn't know and plus like they had a countdown so you could just be like oh, I just missed it that time and it wasn't, because a few times I just generally didn't know the answer and I'd just be like oh I'm not even going to bother guessing I'll just sit here.
I1: OK.
P2: I felt more involved. I felt like I did have to answer.
I1: You had to sort of take part in the whole thing.
P2: Yeah.
I1: So what about if you had the option to, if there was some way in which you could interact with the lecture optionally, so like perhaps submit some questions for the end or something like that, would that be something that you would possibly take part in? So rather than having to go to the lecturer say at the end of it, hey you know, you spoke about such and such a thing and I wanted to ask you, you could send in, you know, like a text or something to a system.
P1:  I would prefer that.
I1: would that be something that would be --?
P1: Yeah because I know sometimes like we had the option of like writing them down but you'd be a bit like oh I don't have time to like write it and the go hand it in so you'd kind of just go oh I'll just leave it and do it later the you completely forget. 
I1: Whereas texting is --.
P1: Texting is easy, it's just straight there.
I1: Is far quicker than --.
P1: Yeah it is. 
P2: I don't think I've ever had the urge to ask a question.
Everyone laughs
P2: But I think if I could text in --.
I2: It's because you Google everything.
P2: Yeah, probably, but if I ever did need to ask a question I probably would prefer to text in than just go to the front and just awkwardly stand there and be like, excuse me.
I1: OK. 
I2: Would you prefer texting to just writing on … and just putting it in a box or something?
P2: Yeah, yeah.
I1: Because I know that [lecturer], for our lectures that we had with her she had like a system where she handed out some little cards and we wrote anything on and put them in  box but you don't particularly like that approach or you would just prefer some sort of electronic version?
P1: I prefer electronic because nobody necessarily needs to know what you've asked because sometimes you like, if everyone's like oh its really simple and your sat there like well I don't find it's kind of, I'd be a bit embarrassed to go ask if I didn't understand and everyone would be like oh, go throw it in the bowl, as if you don't know that really.
I2: Even if it it's just on a piece of paper that you quickly scribble on and pop in a box you'd still feel a bit of pressure?
P1: Yeah because it's like, sometimes they do like announce the next lecture and people bring it up but it's rather -- if it was text and it was like one to one I'd prefer it kind of that way.
I1: So what if like if the end of the lecture the questions asked came up, like on a screen or something, and then they just quickly talked through them, would that be something --?
P1: I don't think I'd mind because then people still wouldn't know but the writing there's still that opportunity.
I1: There's a possibility for people around you to see it, I understand that.
I2: So do you think you'd be more or less likely to ask a question if it was -- what am I trying to say -- if it was a choice between writing a question and just not asking the question which one would you choose?
P1: I'd rather not ask.
I2: You would rather not ask, but when texting comes in there --.
P1: I'd do it more likely, because if I don't understand something I'm more likely to like ask a friend text and if they don't know I'd just kind of leave it and never know but if there was a text option then I'd probably do it that way.
I1: [to P2] you presumably just Google whatever --.
P2: Yeah, just Google it, but no I would rather -- if it was texting or asking or writing it out I'd probably just ignore it. 
I2: You think you'd be more likely to not ask a question at all if it involved writing it down than texting it in.
P2: I feel like if it wasn't described fully in the lecture then it probably is not as vital as another -- as something else that may pop up.
I1: OK, that's an interesting way to look at things [long pause] I think we've asked pretty much everything that --.
I2: Have you got anything else that you'd like to say about it?
P1: I just know that on a few of ours when like the program wasn't working it did kind of like, we'd have to resort to like the putting your hands up kind of thing [laughs], it was just a bit annoying when the program wouldn't work with certain computers and you spend most of the lecture trying to get it to work.
I2: OK, so that's a potential draw back.
P1: Yeah because it was quite annoying when like there'd be like, three lecture in a row, they'd be like right we're going to do clickers and then it wouldn't work and you'd just be like I don't want to do it anymore because it's just annoying when you're just sat there waiting for it to work.
P2: I can't really think of anything…can I delete the app now? I actually still have it.
Everyone laughs
P2: I didn't know whether it was going to creep up again.
I2: [jokingly] well you know, it might be coming in full swing next year so --.
P2: Well I might as well keep it then.
I1: Yeah it might be useful next year, you never know.
I2: So generally would you like to see more clickers in more lectures?
P1: I would yeah
P2: I think it's module specific. 
I2: You think it depends on module?
P2: Yeah, for [module A] which was the one it was tested on that was -- it was a good thing to use it for because there was a lot of places where it'd be good to ask questions and they were like quick ones but like for one of my other modules it'd be too strenuous.
I2: What module is that?
P2: The [module B], it'd probably be a bit more like oh, because it's a lot more confusing, there's a lot of stuff in there where like it's a little bit hard to put a little quick question, a quick quiz in.
I1: Because the content is more sort of in depth?
P2: Yeah, I can't think of anywhere in the lecture where apart from right at the beginning where you would need to ask questions because by the time you get in depth some of the stuff's too confusing anyway before people start asking you questions if you've not had time to, collect it, sort of thing.
I1: Yeah I did the module so I kind of understand where you're coming from there. I know what you mean but putting it into words is a little bit difficult. 
I2: So, you've said that obviously there's a bit of a battery issue but you still prefer phones to actual clickers then, you'd still say that was the case yeah?
P1: Yeah.
Interviewers confirm there are no more questions and everyone is happy to finish the discussion before terminating the recording. 


Interview 2: June 2014
Participants: Interviewer (noted by Q), 8 participants. Due to background noise and the larger group size, it was not possible to accurately identify individual contributors.
Q: Did you all use the clickers/phones? 
Yes
Q: Did anyone not use it?
No.
Q: Did you all use your own phones? 
Yes
Yes
Yes, we did.
The first time we didn't.  Yes, the test to start with and then we used our own phones.
There was an app.
Q: Would you prefer to use your own phone or a clicker?
No, I didn't mind either way.
They didn't work, it wouldn't load.  It took ages to log on in the first place as well.
Q: So would you say that was one of the main barriers to using them?
And obviously if you were a person who had trouble with it.
It relied on wi-fi didn't it?  So it wasn't…
University wi-fi is not the most reliable.
It can be a bit sketchy at times.
Whereas the clickers were obviously in the same room just using them…
Q: If you were asked to use your phone for interactive lectures and there was no clicker option would you mind?
No, I don't mind but I wouldn't… but I would prefer to not do it.
If we had to use the phone then yeah.
If you want us to join in then give us a clicker but it's fine otherwise to use your phone.
Q: Would you be willing to pay to use your phone or use up some of your allowance to send text messages if there was no wi-fi available?
No.
No.
Only if we get it free.
Q: Distraction – do you use your phone/tablet/laptop in lectures normally?
Do you mean do we use it for the lecture or use it in general?
Use it in general?
Q: In general…
Yes.
No.
It depends.
Q: When are you most likely to use it in lectures?  Many lecturers don't like you using your phone in a lecture, how can we best stop you from using your phone?
Keep you engaged.
I found that when the clicker thing was on I didn't use it as much because when the app was open on the clicker system I didn't want to lose connection and then not be able to log back in.
Q: So when the clicker was actually on…
I didn't use it as much.
I don't think… I think whatever you do there's always going to be people who will check Facebook in lectures.  I don't think you're ever going to stop them.
Especially the two hour lectures.
Especially with the free wi-fi.
When it works.
Q: Do you think that having to use your phone made you more likely to use it for other purposes?
I don't think it makes a difference at all.
No.
Q: It doesn't matter if you've got permission or not? You'd still use it?
Especially not in big lectures, there's lot of people.
Q: What do you use it for?
It depends.
Sometimes you're not doing things for the lecture but you're checking your email or eBridge or something like that.
Doing a Tesco shop.
I have done online shopping.
I haven't.
Q: Of the time you've spent on the Internet during lectures how much is work-based and how much is socialising?
It depends on the lecture, every one is different.
It depends on how interesting the lecturer is.  Some of them you're sat there like… “I don't get any of this”.  
Sometimes it gets too complicated and you switch your brain off and go in a daze.  Come back to it when you're in a better frame of mind.
I'll tell you who's the best at keeping your attention – [lecturer] because she picks on you randomly dead quick so I tend not to do it with [lecturer] …
Because she'll pounce on you.
Yes.
She's got eyes like a hawk.
Some lecturers drone on and don't really pay much attention to what anyone else is doing, they just get their lecture done.
It all depends on the lecture theatre.
Yes, it does.
Which is the awful one?  Is it [lecture theatre]?
It's really horrible.
It's not a good one. I hate that.
I don't like it.
It echoes.
I'm more likely to use my phone in there.
I can't concentrate when I'm in [lecture theatre].
It's like being told not to use your phone, so you would use your phone.
No, I wouldn't like to use my phone with the app.
Q: What kind of work-based stuff do you do, if you do it on your phone/tablet?
Check eBridge results definitely.
Check assignments.
Sometimes on my iPad I just go through my slides.
If they've moved off it too quickly or you've missed what they've said.
Q: You find it useful for that?
Yes, you can go over it.
Yes.
Q: Do you think you are distracted by using the Internet?
Yes.
In some ways, yes.
Definitely.
I don't think I'm distracted by it.  I tend to turn to it when I'm bored or not engaged with lectures.  I wouldn't think of my phone going off in my pocket and check it and that distracts me… I think if I've lost interest in the lecture then get my phone out and start doing something. So I wouldn't think of it as a distraction as such.  Because if it wasn't my phone I'd just doodle in my notepad.
It depends on the lecture slides and following the lecturer if they go off, you don't know what they're talking about.  You go off on the Internet.
If I was interested, like some lectures I will sit from start to finish completely engaged in it all and listen as much at the end as I did at the start, it depends… it depends on what you're interested in.
Q: Can you multi-task during lectures?
Not very well.
No.
I can multi-task in a lecture.
Q: Does it matter if you're distracted?
Yes.
Well…
It depends by what.
To some extent.  Like if someone else is distracting you then that's because you're obviously not paying attention to the lecture and you miss key points.
Yes.
I think it depends on who the lecturer is though.  Some lecturers literally read off the slides so if you were distracted it wouldn't matter because you could look back at the slides or if you have a Dictaphone you could just listen back to it anyway.  So being distracted within the lecture isn't so much of a deal if you can then go back to it and read it later and do wider reading.
I think it's up to yourself as well isn't it?
Yes.
We're adults so it's up to you like how much you put in to your lecture. I don't think it all boils down to the lecturer.
You can go over the lecture afterwards.
Q: Is work or social stuff more distracting?
Social side.
Social.
Facebook.
I think that's a really broad question.
I know, because if you've got deadlines a lot of people miss lectures because they've got deadlines.  A lot of people choose not to go to a lecture because they're finishing work or whatever else.
They're finishing assignments.
Actually I would think less of missing a lecture if it was for a Uni-based thing I wouldn't feel so guilty about it as such. I would think it was okay as I need to do this. So in that respect – work.
Half the lectures you can just read in your spare time.
Yes, a lot of people do that.
Q: Are you distracted by other people socialising or looking at work stuff?
Socialising – no.
Do you mean like talking or doing social things?
If they're on the phone it's not distracting.  But if they're talking then it will distract me.
People sometimes are playing games and that's distracting.  You can see it out of the corner of your eye.  Things like that.  That's distracting.
I think I get distracted by people talking because certain people sit behind me and it's distracting.
Q: Do you know how to switch off notifications on your device? (everyone nodded)
Yes
Yes
Yes
Q: Are you distracted when you receive notifications?
Not really no.
No.
Q: It doesn't make you think “I'll have a look at that?”
No.
It depends what it is.
Q: Do you think that using your phone or clickers had a positive effect on your learning?
Not really.
I don't think it made a massive impression.
It was alright.
It made you think a bit more in the lecture instead of just writing it down.
You had to think of the answer.
You could be just like “whatever” and press the button because it's anonymous isn't it so there's no come-back on you whereas if a lecturer picked you out and asked you a question then you have to focus and answer it whereas with the thing you can just …
I think it's because it's anonymous it makes you want to answer more.  If you get it wrong you're not going to get shamed.
Yes.
You're not going to look daft.
You're not going to feel dumb if you get it wrong.
It's just when they put the lectures later on eBridge they leave the clicker side it's always zero percent.  So unless you know the answer but you should know the answer, but if you don't know looking through the slides what the answer is.
They're not so good for revision in that respect.
It doesn't show the answer.
Q: It should show the answer?
Yes, that would help.
Q: When revising did you find that you remembered clicker topics better or worse than topics not covered with clickers?
Not really.
It didn't make a difference.
Sometimes it wouldn't give the right answer on the clicker question which was really annoying.  They'd give the graph of who answered what … and you wouldn't get the answer and that wasn't helpful at all it was just the people answering in various ways.
I don't think it's a massive breakthrough.
I mean there and then it's good and it makes you think but I'm not sure if it has a lasting impression.
No… I couldn't say I could sit and tell you I answered this and it was that.
I think lectures work better when they're seminar based and you have to have a conversation about a topic.
Workshops and seminars…
We don't get many of them.
We just kind of get lectures.
Q: Do you think they're more helpful?
Yes, instead of having lectures, have seminars that would be like ideal.
Every week in foundation year we had a seminar which I found loads more helpful.  Because we had the question/answer part seminar and then you had a short class test at the end of each one which was worth 5% of the module or something but the grades were much better because you had to engage and you had to know it at the end of the lecture and I found that a hundred times more useful than the clicker things.  It definitely was better and I think it would work with large classes because it was just a really short question answer session sheet at the end of the lecture so you had to absorb the lecture to be able to get a mark to pass and if you did and you paid attention you could potentially get 100%.  So I thought that was miles more useful than the clickers.
Who was that for?
[lecturer] did it, [lecturer] did it and [lecturer] did it.  It was really good that.
Q: Did the interactive questions help with your revision?
No.
No.
Q: So if it recorded the score…?
Yes, it would be helpful.
Oh yes.
Yes, you could look at it afterwards.
Q: Is having access to the Internet during lectures generally helpful or detrimental to your learning?
More detrimental to be honest, like I will go on Facebook…
I do think having wi-fi available at University is helpful.
It is helpful but at the same time it can be abused and not be used properly.
It depends how it's used.
It depends on the person.
Sometimes if the lecturer says something it sparks something an interest you can…
…look it up.
I think so.  I wouldn't appreciate the facility being removed.
No.
Because we're adults aren't we.  It's up to us to make the decision.
It's our responsibility.
Q: I suppose it depends how it's used in the lecture?
Yes.
Q: Generally would you like more lecturers using clickers or phone apps?  Do you have a preference?
Between the two or not at all?
Q: Would you just like to see more of them or not at all?
Not really.
No.
If it was, it would be clickers, not mobile.
Clickers.
If it were a phone app then clickers yes.  Because you put some people at a disadvantage if they don't have a smartphone or whatever they're at a disadvantage.
It's like the foreign exchange students often just have a cheap pay as you go phone so…
And also I did find it quite good but in a lot of the lectures they didn't work.  In [lecturer]'s particularly.  She spent five or ten minutes of the lecture trying to make the clicker work instead of getting on with the lecture.
I think it's like a small gimmicky thing every now and then it's got its place but I wouldn't like it more than that.
Q: If they used it in all lectures?
No I wouldn't like that.
Q: Any other opinions?
Some lecturers, a certain person, some lecturers do lectures that are too long.  We don't get a break.  And sometimes even an hour it can get quite heavy going.
I think that's really valuable actually, having a little break in between.
It's like with me, my sugar levels drop and my concentration… I fall asleep.
Q: I suppose if it's an intensive one?
I'm just like, in hindsight, if it's a two hour lecture, by the end you're watching the clock, you're not going to look back on it as an enjoyable experience.  I think you retain things better if you think 'I really liked that'.
[lecturer]'s lectures – the half hour in between makes it much easier to concentrate.
If you made lectures more attractive, broke them up a bit more.  I know people who just don't do extra reading – if you did more things like that, you'd get more out of your course.  If you had to learn stuff before the lectures you'd feel a bit more pressure, plus if the lectures are fun.
I think if there was a bit more back and forth between you and lecturer, it makes them a bit more personal, a bit more approachable and you feel like you can ask a question at the end.  Like if you're actually interacting with them.  Like I've noticed the difference between Hull [campus] and Scarborough [campus] and the relationship between the lecturers at Scarborough and their pupils, I know it's smaller but they're a lot more familiar with them.  And you can ask them more questions and you learn more.  
They're definitely more available and more enthusiastic to help out.
It depends on how lecturers go about teaching.  Some lecturers are more introvert and others make more of an effort.  Some lecturers need to make more of an effort.
They're academics though aren't they?  They're only there because they're interested in [research area]… and they do lecturing on the side.  Some.  Not all of them.  Some.

Interview ends
