# Supplementary material for: Interactive lectures: Clickers or personal devices?
Source: F1000Res. 2015 Mar 12;4:64. [Version 1] doi: 10.12688/f1000research.6207.1 (PMC4648207; doi:10.12688/f1000research.6207.1)
Supplement: Peer review history

# Peer review: Referee response for version 1

**DOI:** 10.5256/f1000research.6656.r9033

**Published:** 2015-06-18

**Authors:** Chris Buddle

**Affiliations:** 1 Department of Natural Resource Sciences, McGill University, Quebec, Canada

This is a nice study that focuses on a very interesting question - about whether students use their own devices differently than 'traditional' clickers (or student response systems). The article is well written and covers the topic well. The results are well presented, convincing, and this is a valuable contribution.

The authors may want to weave some additional literature into their paper, e.g., I did come across this overview of a recent paper that shows how texting and tweeting during lecture could be 'beneficial' to students. It certainly aligns with my own thinking on the topic. See: https://www.timeshighereducation.co.uk/news/could-texting-and-tweeting-lectures-be-good-learning

I might also suggest the authors discuss a little more about how important the role of the instructor is in the process of using any kind of 'interactive' technology in a classroom. So much depends on the level of comfort of the instructor, and the 'distracted' element of the student responses is related to this. I assume that the level of distraction in this case study is relatively low because the instructor is being a good role model and providing a rich learning environment. The challenges when an instructor shoehorns in "clickers" (or devices) without adjusting lecture style, etc. is significant and should be addressed.

It's a little unfortunate that the authors did not have a true control - ie., no devices or clickers. However, I also realize this is very difficult to achieve in a teaching and learning context, and when student grades are on the line. However, it does raise the issue of how general the findings might be, based on this case study, and perhaps some additional text to reflect this could be inserted.

Overall, however, this is a very good case study and is a good contribution!

I have read this submission. I believe that I have an appropriate level of expertise to confirm that it is of an acceptable scientific standard.

# Peer review: Referee response for version 1

**DOI:** 10.5256/f1000research.6656.r7938

**Published:** 2015-04-20

**Authors:** Alan Cann

**Affiliations:** 1 Department of Biology, University of Leicester, Leicester, UK

This interesting and timely study raises intriguing questions for future work. While it clearly shows that interactive lectures are popular with many students, there is some evidence of a cohort who prefer their lectures to be free from technology distractions. As the authors correctly note, the big unanswered question here is the potential impact on student attainment, which, as with most technological interventions, is difficult to measure accurately. Nonetheless, this work represents and responds to an area which many universities and staff are currently pondering, and as such it represents a useful contribution. I have no comments on the methodology, statistical analysis or writing which has been carried out at an exemplary level, other than to say it would be useful to see effect sizes calculated where statistically significant differences are claimed.

I have read this submission. I believe that I have an appropriate level of expertise to confirm that it is of an acceptable scientific standard.
